# ER Stress in Retinal Degeneration in S334ter Rho Rats

**DOI:** 10.1371/journal.pone.0033266

**Published:** 2012-03-14

**Authors:** Vishal M. Shinde, Olga S. Sizova, Jonathan H. Lin, Matthew M. LaVail, Marina S. Gorbatyuk

**Affiliations:** 1 Department of Cell Biology and Anatomy, University of North Texas Health Science Center, North Texas Eye Research Institute, Fort Worth, Texas, United States of America; 2 Department of Pathology, University of California San Diego, San Diego, California, United States of America; 3 Department of Ophthalmology, Beckman Vision Center, University of California San Francisco, San Francisco, California, United States of America; Massachusetts Eye & Ear Infirmary - Harvard Medical School, United States of America

## Abstract

The S334ter rhodopsin (Rho) rat (line 4) bears the rhodopsin gene with an early termination codon at residue 334 that is a model for several such mutations found in human patients with autosomal dominant retinitis pigmentosa (ADRP). The Unfolded Protein Response (UPR) is implicated in the pathophysiology of several retinal disorders including ADRP in P23H Rho rats. The aim of this study was to examine the onset of UPR gene expression in S334ter Rho retinas to determine if UPR is activated in ADRP animal models and to investigate how the activation of UPR molecules leads to the final demise of S334ter Rho photoreceptors. RT-PCR was performed to evaluate the gene expression profiles for the P10, P12, P15, and P21 stages of the development and progression of ADRP in S334ter Rho photoreceptors. We determined that during the P12–P15 period, ER stress-related genes are strongly upregulated in transgenic retinas, resulting in the activation of the UPR that was confirmed using western blot analysis and RT-PCR. The activation of UPR was associated with the increased expression of JNK, Bik, Bim, Bid, Noxa, and Puma genes and cleavage of caspase-12 that together with activated calpains presumably compromise the integrity of the mitochondrial MPTP, leading to the release of pro-apoptotic AIF1 into the cytosol of S334ter Rho photoreceptor cells. Therefore, two major cross-talking pathways, the UPR and mitochondrial MPTP occur in S334ter-4 Rho retina concomitantly and eventually promote the death of the photoreceptor cells.

## Introduction

Retinitis pigmentosa (RP) is an inherited retinal disorder that is caused by the progressive loss of rod and cone photoreceptors with clinical hallmarks that include sensitivity to dim light, abnormal visual function and characteristic bone spicule deposits of pigment in the retina [1]. This disease affects approximately 1 in 3200, and an estimated 1.5 million people are affected worldwide. The autosomal dominant form of RP (ADRP) is associated with mutations in at least 14 different genes; however, mutations in the rhodopsin gene (*RHO*, OMIM 180380, accession ID U49742) are the most prevalent mutation identified to date resulting in 30% to 40% of all ADRP cases [2], [3].

S334ter rhodopsin (Rho) rats (line 4) express rhodopsin gene containing an early termination codon at residue 334 and is a model of a number of Rho truncation mutations in human RP patients (http://www.ask.com/wiki/Retinal_Degeneration_(Rhodopsin_Mutation). This mutation results in the expression of a rhodopsin protein that lacks the 15 C-terminal amino acids that are involved in rhodopsin trafficking to the photoreceptor outer segments and in the deactivation of the rhodopsin protein after light absorption. A previous study conducted using this rat model demonstrated that the nature of the rhodopsin sorting defect, but not the constitutive activation of the phototransduction cascade, contributes significantly to apoptosis by interfering with the normal cellular machinery in the post-Golgi transport pathway or in the plasma membrane [4]. The study also revealed a correlation between the severity of mis-sorting of the truncated rhodopsin protein and the rate of cell death in these animals. Although the primary cause of degeneration in the S334ter-4 Rho photoreceptors has been identified, the precise mechanism responsible for triggering the apoptotic cascade remains unknown.

The apoptotic death of photoreceptor cells is the cornerstone of the pathophysiological process in RP [5], [6]. Although the role of caspases in the execution of apoptosis in retinal degeneration has been demonstrated, the process has not been fully investigated. Moreover, conflicting data has been published regarding the pro-apoptotic cellular signals that lead to the deterioration of photoreceptor cells. These studies indicate that diverse cellular pathways are involved in the demise of photoreceptor cells; for example, two independent caspase activation pathways (the cellular stress response and the death receptor pathway) have been identified in the rd mouse model [7]. The Bid protein, which is activated by caspase-8, and phosphorylated p38 MAPK play a key role in the cross- talk between these two activation pathways resulting in the release of cytochrome *C* and the activation of casape-3 in these animals. However, a separate study of the rd mouse suggests that cell death occurs through a caspase-independent pathway, and the DNA cleavage originates independently of caspase-9, -8, -7, -3, and -2 activation and cytochrome *C* release [8]. Another study demonstrates that in S334ter-4 Rho retinas, apoptosis contributes to the progression of retinal degeneration in these animals [9], [10]. Moreover, caspase-dependent signaling involving the activation of caspases-3 and -9 and cytochrome *C* leakage accompanies the activation of calpains and poly (ADP-ribose) polymerase (PARP) and the down-regulation of calpastatin [10].

Recently, it has been proposed that the death of RP photoreceptors may involve multiple mechanisms, including caspase-dependent and caspase-independent pathways. Due to their energy production and calcium homeostasis properties and their ability to compartmentalize cell death activators, mitochondria play a central regulatory role in this process [11], [12]. In addition, the activation and translocation of AIF (Apoptosis-inducing factor) from the mitochondria and the translocation of caspase-12 (ER stress-associated caspase) to the nucleus in dying photoreceptors suggests that there is a link between the mitochondrial caspase-independent pathway and the endoplasmic reticulum (ER) stress signal in the cytoplasm [12]. These studies suggest that the ER stress response is involved in the pathological events of ADRP photoreceptors. Therefore, it will be important to determine if mislocalized truncated proteins, as opposed to misfolded truncated proteins, are involved in the retinal pathology of S334ter-4 Rho rats.

Recently, many studies have examined the involvement of the ER stress response or the unfolded protein response (UPR) in retinal degeneration [13], [14], [15], [16], [17]. In a previous study, we suggested that the ER stress response is involved in ADRP progression in P23H Rho-3 transgenic rats and that over-expression of the Bip/Grp78 protein reprograms the ER stress signal and protects the P23H Rho photoreceptors from degeneration [16]. Although the investigation of the ER stress response in P23H Rho photoreceptors in this ADRP model is not complete, the activation of the ER stress response in the S334ter-4 Rho rat model has never been analyzed in detail. Therefore, we investigated how the UPR contributes to the degeneration of S334ter-4 Rho photoreceptors. Our interest has been fueled by a previous study in S334ter-4 Rho rats demonstrating that the truncated S334ter-4 rhodopsin protein is retained in the cytoplasm or is associated with the cell membrane [4], suggesting that the accumulation of truncated rhodopsin in the cytoplasm may overwhelm the protein degradation system. In addition, we are interested to determine if the activation of the ER stress signal in this model initiates apoptosis and if there is a dynamic link between the activation of the UPR and the UPR-associated and mitochondria-induced apoptosis.

Therefore, the purpose of this investigation was to determine whether the ER stress signaling is activated upon S334ter-4 Rho photoreceptor degeneration and to demonstrate that additional cellular pathways, such as mitochondria-induced apoptosis, occur in S334-4ter Rho photoreceptors to initiate the collapse of S334ter-4 Rho photoreceptors during the progression of ADRP.

## Results

The retinal degeneration in S334ter-4 rats has been described previously [4], [18], [19], http://www.ucsfeye.net/mlavailRDratmodels.shtml. Briefly, this is a degeneration of photoreceptors of moderate rate compared to other rodent models. Photoreceptor degeneration begins at about postnatal day (P) 13 [4] and about 25% of the photoreceptors are lost by P30, 50% by P60 [19] and about 65% by P120. In addition, high resolution light microscopy has demonstrated that at P4, P6, P8 and P10 the S334ter-4 retinas are indistinguishable from age-matched wild-type controls and this is reflected in a normal ONL thickness at P10. A very few pyknotic nuclei are observed at P12 resulting in the ONL thinning only beginning at P21 (LaVail, unpublished observations). Therefore, we choose P10 as the first time point to observe the kinetics of the gene expression involved in the UPR and UPR-associated signaling pathways.

Previous studies have demonstrated the cytoplasmic localization of the S334ter-4 rhodopsin protein in the retina [20] of the transgenic lines 3 and 5. In our study of S334ter-4 Rho, we confirmed the mislocalization and retention of truncated rhodopsin in the cytoplasm of S334ter-4 Rho photoreceptors (data not shown). Therefore, we were interested to determine if mis-trafficking of the S334ter-4 Rho protein provokes the ER stress signal, and if the activation of ER stress correlates with the expression of genes that modulate the redox potential of ADRP photoreceptors and affect homeostasis in the ER.

### Expression of genes associated with oxidative stress is elevated in S334ter-4 Rho retinas

ER homeostasis is a fragile equilibrium that is modulated by dysregulation of the calcium or oxidative/reductive balance, features that have previously been associated with oxidative stress. Recently, it has been demonstrated that two processes, UPR activation during oxidative stress [21], [22] and raised oxidative toxicity due to the involvement of ER stress, are linked [23]. Therefore, we analyzed the relative expression of genes that are sensitive to an oxidative/reductive environment.

**Figure 1:**
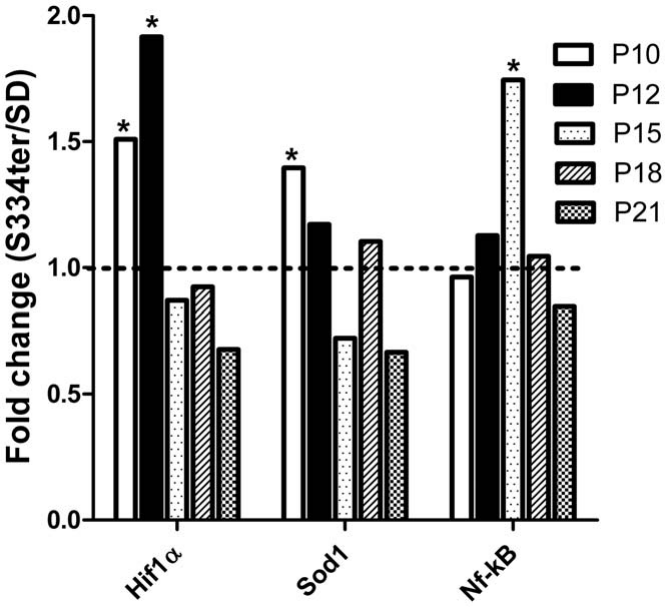
Relative expression of the oxygen stress-induced Hif1a, Sod1 and Nf-kB genes in S334ter-4 Rho retinas at different ages. Relative gene expression in S334ter-4 Rho retina was measured on P10, P12, P15, P18 and P21 and a fold change was expressed as a ratio of S334ter-4Rho relative expression to SD relative expression. Expression of the Hif1α and Sod1 genes was significantly induced in P10 S334ter-4 Rho compared to SD retinas (1.5- and 1.4-fold, respectively, *P*<0.05, *). On P12, increased Hif1a expression was still evident (1.9-fold, *P*<0.05,*); however, the Sod1 expression was decreased compared to P10. When compared with Hif1a and Sod1 mRNA, the relative accumulation of Nf-kB mRNA in S334ter-4 Rho retinas exhibited alternate dynamics: a 1.7-fold increase in Nf-kB expression was observed on P15 only (*P*<0.05,*), whereas the Hif1α and Sod1 expression was diminished on P15.

To further assess the effect of the mis-localized truncated rhodopsin protein on oxidative stress, we examined the expression level of the transcription of hypoxia-inducible factor α (Hif1αa), superoxide dismutase (Sod1) and the nuclear factor kappa-light-chain-enhancer of activated B cells (Nf-kB) genes that are known to modulate the redox state of the cell. Figure 1 shows the relative expression of these genes in S334ter-4 Rho and SD (Sprague Dawley) rats on P10, P12, P15 and P21.

Our data demonstrated that Hif1α gene expression was upregulated 1.5- and 1.9-fold on P10 and P12, respectively (0.93±0.06 in SD vs. 1.4±0.17 in S334ter-4 Rho and 0.62±0.09 in SD vs. 1.2±0.14 in S334ter-4 Rho, respectively, *P*<0.05).

On P15, the expression level of Hif1α dropped dramatically compared with that observed in the SD retina. The expression of Sod1 also increased dramatically (1.4-fold) (0.69±0.04 in SD vs. 1.334±0.1 in S334ter-4 Rho, *P*<0.05), but this increase occurred on P10. SOD1 expression subsequently declined to the level of the SD over time. In contrast, Nf-kB gene expression increased 1.7-fold (0.9±0.18 in SD vs. 1.6±0.2 in S334ter-4 Rho, *P*<0.05) only on P15. At other time points, the Nf-kB gene expression was comparable to that in the wild-type (WT) retinas.

### Comparative analysis of ER stress and ERAD-associated genes in S334ter-4 Rho and SD retinas

**Figure 2:**
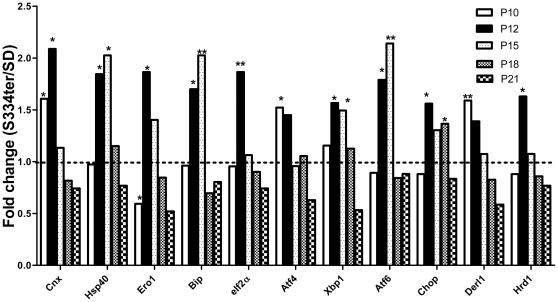
Relative expression of ER stress- and ERAD-related genes in S334ter-4 Rho retinas. The UPR gene expression was altered in S334ter-4 RHO retinas. Relative gene expression in S334tr Rho retina was measured on P10, P12, P15, P18 and P21 and a fold change was expressed as a ratio of S334ter-4Rho relative expression to SD relative expression. On P10, there was a significant reduction in the Ero1 gene expression suggesting that the ER homeostasis in S334ter-4 Rho retinas is imbalanced. The expression of this gene is decreased 2-fold (*P*<0.05, *) in S334ter-4 Rho retinas compared with SD retinas on P10. The increased expression of Calnexin (Cnx) and Atf4 genes are the first ER stress markers that respond to ER disturbance. The expression of these genes was increased 1.6- and 1.5-fold, respectively, (*P*<0.05, * in each case) on P10. On P12, the expression of other UPR upstream and downstream markers, such as Hsp40/Dnajc10, Ero1, Bip, eIf2a, Xbp1, Atf6 and Chop, were detected, which was indicated by relative increases of 1.8-, 1.8-, 1.5-, 1.9-, 1.6-, 1.8- and 1.6-fold, respectively, (*P*<0.05, * in each case with the exception of eIF2a where *P*<0.01, **). On P15, the Cnx, Ero1, eIf2a, Atf4 and Chop genes were expressed to a lesser extent or there was no significant difference in their expression levels in S334ter-4 Rho retinas compared with SD retinas. However, the Hsp40/Dnajc10, Bip, Xbp1 and ATf6 mRNAs were significantly induced on P15. Their relative expressions were 2-, 2-, 1.5- and 2-fold higher in S334ter-4 Rho retinas compared with the WT group (*P*<0.01 for Bip and Atf6, and *P*<0.05 for Hsp40/Dnajc10 and Xbp1). On P21, the expression of all genes was insignificant in the S334ter-4 Rho retinas compared with the SD retinas. Derl1 and Hrd1 gene expression was upregulated 1.5- and 1.6-fold on p10 and p12, respectively (*P<*0.05 in each case).

Figure 2 demonstrates the results of RT-PCR analysis of the genes involved in the activation of ER stress signaling in S334ter-4 Rho rats. On P10 in transgenic retinas, calnexin (Cnx) and ATF4 gene expression was significantly upregulated 1.6- and 1.5-fold, respectively (0.9±0.05 in SD vs. 1.4±0.2 in S334ter-4 Rho and 0.9±0.04 in SD vs. 1.3±0.1 in S334ter-4 Rho, respectively, *P*<0.05 in both cases). Furthermore, Ero1 gene expression was downregulated 2-fold (1.2±0.18 in SD vs. 0.6±0.18 in S334ter-4 Rho, *P*<0.05). On P12, the number of genes involved in the ER stress response was extended and included the following: Cnx (2.1-fold increase; 0.62±0.09 in SD vs. 1.3±0.16 in S334ter-4 Rho, *P*<0.05), Hsp40/Dnajc10 (1.8-fold increase; 0.65±0.1 I SD vs. 1.21±0.18 in S334ter-4 Rho, *P*<0.05), Ero1 (1.8-fold increase; 0.8±0.13 in SD vs. 1.5±0.2 in S334ter-4 Rho), Bip (1.7-fold increase; 0.7±0.01 in SD vs. 1.2±0.1 in S334ter-4 Rho, *P<*0.05), eiF2a (1.9-fold increase; 0.80±0.13 in SD vs. 1.50±±0.2 in S334ter-4 Rho, *P*<0.01), Xbp1 (1.6-fold increase; 0.72±0.08 in SD vs. 1.17±0.13 in transgenic rats, *P*<0.05), Atf6 (1.8-fold increase; 0.64±0.09 in SD vs. 1.15±0.13 in S334ter-4 Rho, *P<*0.05) and Chop (1.5-fold increase; 0.8±0.05 in SD vs. 1.3±0.12 in S334ter-4 Rho, *P*<0.05). On P15, the expression of Cnx, Ero1, eIF2a and Atf4 dropped to different extents, and although in some cases, the expression level of these genes was higher than in the SD retinas, the relative expression of Chop protein was consistently 1.3-fold higher than in the SD retinas. On P15, the relative expression of the following genes was significantly elevated: Hsp40/Dnajc10 (2-fold increase; 0.6±0.12 in SD vs. 1.2±0.22 in S334ter-4 Rho, *P*<0.05), Bip (2-fold increase; 0.65±0.12 in SD vs. 1.32±0.16 in S334ter-4 Rho, *P*<0.01), Xbp1 (1.5-fold increase; 0.9±0.16 in SD vs. 1.36±0.17 in S334ter-4 Rho, *P*<0.05), Atf6 (greater than 2-fold increase; 0.60±0.17 in SD vs. 1.22±0.073 in transgenic rats, *P*<0.01). On P18, the expression levels of all genes analyzed dropped to the level of the SD retinas. The exception to this finding was the Chop gene, which exhibited upregulated expression in P18 retinas, and the Xbp1 gene, which was significantly downregulated in P18 S334ter-4 Rho retinas. Therefore, the data confirmed that the expression of ER stress-related genes, the eiF2a and Atf4 genes (the PERK pathway), the Atf6 gene (the ATF6 pathway) and the Xbp1 gene (the IRE1 pathway) were upregulated in P12–15 S334ter-4 Rho retinas.

We also examined ERAD (ER associated degradation) genes, such as Derl1 and Hrd1. Derl1 participates in the retrotranslocation of misfolded proteins into the cytosol where they are ubiquitinated and degraded by the proteasome. The Hrd1 gene is an E3 ubiquitin-protein ligase and transfers ubiquitin specifically from endoplasmic reticulum-associated UBC1 and UBC7 E2 ligases to substrates, thereby promoting their degradation. The qRT-PCR analysis of these genes in SD and S334ter-4 Rho retinas (Figure 2) showed that both genes were transiently activated on P10 and P12. Derl1 expression increased 1.6-fold on P10 (0.9±0.03 in SD vs. 1.4±0.2 in S334ter-4 Rho, *P<*0.01) and subsequently decreased slightly over time. The expression of Hrd1 increased significantly (1.6-fold) on P12 (0.74±0.07 in SD vs. 1.24±0.17 in S334ter-4, *P*<0.05). We also examined the expression of the Edem1 and Edem2 genes and determined that there was no significant difference in the expression levels in S334ter-4 Rho retinas compared with control retinas.

**Figure 3:**
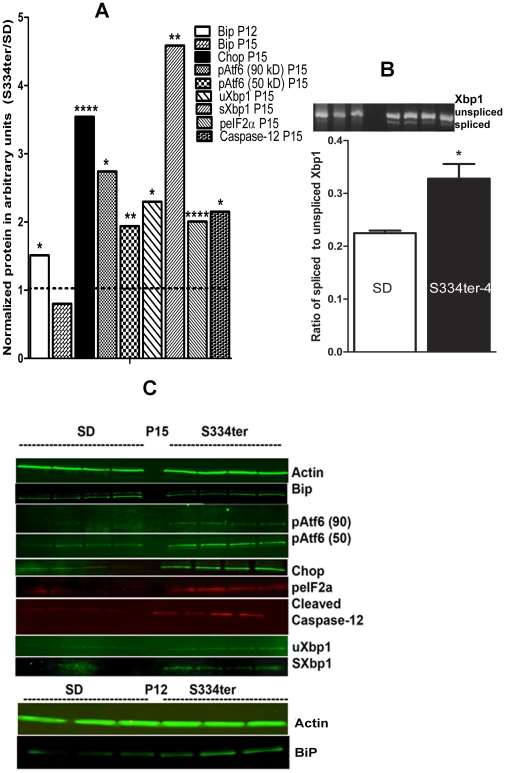
The ER stress markers BIP and CHOP proteins in retinas from S334ter-4 Rho rats. A: Ratios of normalized S334ter-4 Rho BiP, CHOP, pATF6 (90), pATF6 (50), peIF2α ,spliced Xbp1(sXbp1), unsliced Xbp1 (uXbp1), cleaved caspase-12 proteins to corresponding proteins in SD CHOP were used to register the alteration of protein expression. Normalization of all proteins was done by detecting β-Actin protein The ER stress markers BIP protein was a 1.5-fold upregulated in P12 S334ter-4 Rho retinas compared with SD retinas (0.047±0.008 vs. 0.071±0.005, respectively; *P* = 0.01). In P15, the level of BiP protein was significantly reduced and was not distinguishable from SD. The Chop protein was also dramatically over-expressed (3.5-fold) (0.016±0.005 in SD vs. 0.60±0.002 S334ter-4 Rho, *P* = 0.0003) on P15 in S334ter-4 Rho retinas. The full length of pAtf6 protein (90 kD) (the Atf6 pathway) was significantly elevated in S334ter-4 Rho retina by 2.7-fold (0.0017±0.0011 in SD vs. 0.0048±0.0007 in S334ter-4 Rho, *P* = 0.04). The cleaved pAtf6 (50) was significantly elevated by 1.93-fold inS334ter-4 Rho retina (0.18±0.004 in SD vs. 0.035±0.002, *P* = 0.004). The peIF2α protein was also significantly increased in S334ter-4 Rho retina and was 0.001±0.0005 in S334ter-4 Rho vs 0.002±0.0004, *P* = 0.0009. The full length of pAtf6 protein (90 kD) (the Atf6 pathway) was significantly elevated in S334ter-4 Rho retina by 2.7-fold (0.0017±0.0011 in SD vs. 0.0048±0.0007 in S334ter-4 Rho, *P* = 0.04). The N-terminal of the full-length of Atf6, cleaved pAtf6 was elevated by 1.93-fold and was 0.18±0.004 in SD vs. 0.035±0.002, *P* = 0.004. We also observed that the peIF2α protein was significantly increased in S334ter-4 Rho retina and was 0.001±0.0005 in S334ter-4 Rho vs 0.002±0.0004, *P* = 0.0009. The spliced Xbp1 protein was detected in S334ter-4 retina. Its level was a 4.5-fold higher in transgenic retina compared to SD and was 0.022±0.003 in SD and 0.1±0.01, *P* = 0.001 in S334ter-4 rats. Unspliced Xbp1 protein was increased to a lesser extent (2.3-fold) in S334ter-4 rats and was 0.049±0.008 in SD and 0.13±0.001, *P* = 0.034 in S334ter-4. Increase in active caspase-12 was observed in S334ter-4 Rho retina. The level of cleaved caspase-12 (20 kD) was elevated in S334ter-4 Rho rats on P15 compared to control over 2-fold and was 0.05±0.007 in SD vs. 0.12±0.02 in S334ter-4 Rho, *P* = 0.014 on P15. B: Upper panel: Quantification of spliced form of the Xbp1 mRNA (the IRE signaling) detected by RT-PCR reaction. We observed 1.45-fold increased in the spliced form of Xbp1 mRNA in S334ter-4 Rho retina. The ratio of spliced Xbp1 to normalized unspliced Xbp1 mRNA was 0.22±0.0006 in SD vs. 0.033±0.031 in S334ter-4 Rho retina, *P* = 0.025). Image of the agarose gel loaded with RT-PCR product obtained with Xbp1 specific primers is shown in a lower panel. C: Images of western blots treated with anti-Actin, Bip, CHOP, peIf2α, pATF6, Xbp1 antibodies and detected with secondary antibodies and infrared imaging scanner are presented.

We analyzed levels of the main UPR markers such as BiP, CHOP, phosphorylated protein kinase RNA-like endoplasmic reticulum kinase (pPerk), phosphorylated eIF2α (peIF2α), active ATF6 proteins and the Ire-mediated unconventional the Xbp1 mRNA splicing in S334ter-4 Rho rats (Figure 3A, B and C) using western blot analysis and a semi-quantitative RT-PCR and determined that the production of the BiP protein increased 1.5-fold in P12 S334ter-4 Rho retinas compared with SD retinas (0.047±0.008 vs. 0.071±0.005, respectively; P = 0.01. However, by P15 the difference between groups was not detected. The CHOP protein was also dramatically over-expressed (3.5-fold) (0.016±0.005 in SD vs. 0.60±0.002 S334ter-4 Rho, P = 0.0003) on P15 in S334ter-4 Rho retinas. The full length of pAtf6 protein (90 kD) (the Atf6 pathway) was significantly elevated in S334ter-4 Rho retina by 2.7-fold (0.0017±0.0011 in SD vs. 0.0048±0.0007 in S334ter-4 Rho, P = 0.04). The N-terminal of the full-length of Atf6, cleaved pAtf6 was elevated by 1.93-fold and was 0.18±0.004 in SD vs. 0.035±0.002, P = 0.004. We also observed that the peIF2α protein was significantly increased in S334ter-4 Rho retina and was 0.001±0.0005 in S334ter-4 Rho vs 0.002±0.0004, P = 0.0009.The hallmark of the IREI pathway, the spliced Xbp1 protein was detected in S334ter-4 retina. Its level was a 4.5-fold higher in transgenic retina compared to SD and was 0.022±0.003 in SD and 0.1±0.01, P = 0.001 in S334ter-4 rats. Unspliced Xbp1 protein was increased to a lesser extent (2.3-fold) in S334ter-4 rats and was 0.049±0.008 in SD and 0.13±0.001, P = 0.03 in S334ter-4. Images of western blots are shown in Fig. 3 C.

The spliced form of the Xbp1 mRNA was increased in S334ter-4 Rho rats as well. The ratio of spliced Xbp1 to normalized unspliced Xbp1 mRNA was 1.45 (0.22±0.0006 in SD vs. 0.033±0.031 in S334ter-4 Rho retina, P = 0.025). An image of an agarose gel loaded with RT-PCR product obtained with Xbp1 specific primers is shown in Fig. 3B.

### Autophagy is involved in retinal degeneration of S334ter-4 Rho photoreceptors

**Figure 4:**
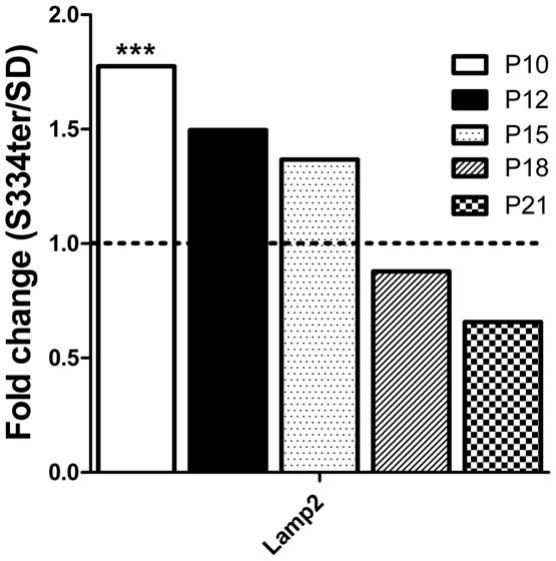
Relative expression of Lamp2 in S334ter-4 Rho retinas. Relative gene expression in S334tr Rho retina was measured on P10, P12, P15, P18 and P21 and a fold change was expressed as a ratio of S334ter-4Rho relative expression to SD relative expression. The Lamp2 gene expression was upregulated in p10-p15 S334ter-4 Rho retinas. In p10, the relative expression of this gene increased 1.7-fold in S334ter-4 Rho retinas compared to that in SD retinas *(P*<0.001), and it subsequently declined in a time-dependent manner.

Knowing that the ERAD genes Derl1 and Hrd1 are transiently upregulated in P10 and P12 retinas, we became interested in investigating the activity of another protein degradation system, autophagy. We examined the relative expression of autophagy hallmark genes, such as Atg5 and Atg7, which are involved in autophagosome formation, and the lysosomal-associated membrane protein 2 (Lamp2). Figure 4 demonstrates that in developing S334ter-4 Rho retinas, the relative expression of Atg5 and Atg7 was not significantly different from that observed in the SD samples. However, the relative expression of the Lamp2 gene was transiently upregulated 1.8-fold on P10 in S334ter-4 Rho retinas (0.86±0.07 in SD vs. 1.5±0.16 in S334ter-4 Rho, *P*<0.01). The expression of this gene subsequently decreased with time. The results of the comparative qRT-PCR analyses are shown in Figure 4.

### S334ter-4 Rho rats exhibit elevated levels of pro-apoptotic gene expression during retinal development

**Figure 5:**
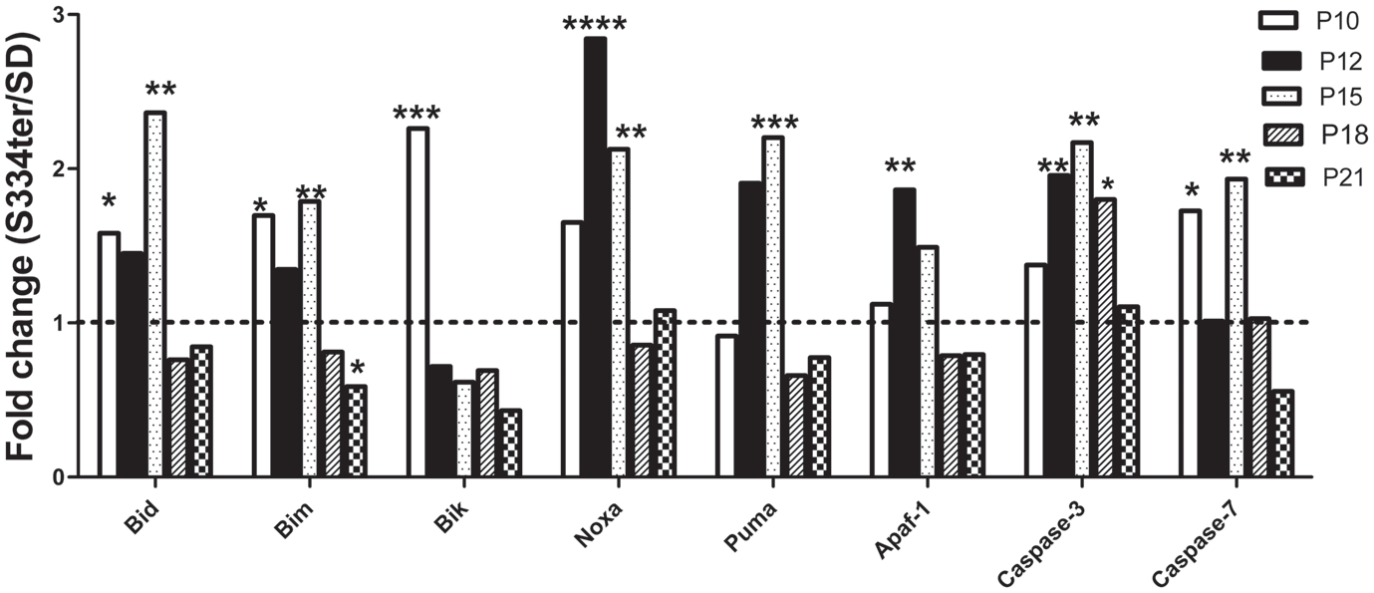
Relative expression of pro-apoptotic genes involved in S334ter-4 Rho retinas. Relative gene expression in S334ter-4 Rho retina was measured on P10, P12, P15, P18 and P21 and a fold change was expressed as a ratio of S334ter-4 Rho relative expression to SD relative expression. The expression of BH3-only proteins, BID, Bim, Bik, Noxa and Puma was upregulated with different patterns in S334ter-4 Rho retinas. On P10, the expression of Bik, Bim and Bid genes increased 2.3- (*P*<0.001) 1.7- (*P*<0.05) and 1.6-fold (*P*<0.05), respectively. The expression of Bim was elevated until p15 and dropped 0.6-fold to WT levels (*P*<0.05) on P21. On P12, the Noxa gene was elevated 2.8-fold (*P*<0.0001) and remained upregulated until p15 (2-fold increase, *P*<0.01) and its expression subsequently dropped. On P15, the Puma gene appears to be upregulated 2.2-fold in S334ter-4 Rho retinas when compared to SD retinas (*P*<0.001). The Apaf1 gene expression was increased in P12 and in P15 retinas. A significant 1.9-fold upregulation Apaf1 expression was detected on P12 (*P*<0.01). The relative expression of caspase-3 was significantly upregulated during the progression of ADRP. On P12, P15 and P18, caspase-3 expression in S334ter-4 Rho retinas was elevated 1.9-fold (*P*<0.01), 2.2-fold (*P*<0.01) and 1.8-fold (P<0.05), respectively. compared to SD retinas. The analysis of caspase-7 gene expression demonstrates a 1.7-fold (*P*<0.05) and 2-fold (*P*<0.01) increase in caspase-7 mRNA on P10 and P15, respectively.

The ADRP photoreceptors degenerate and die through the process of apoptosis. Therefore, we examined the expression of pro-apoptotic genes belonging to the Bcl-2 family: the BH3-only interacting domain death agonist (BID), the Bcl-2-interacting killer protein (Bik), the Bcl2-like 11 apoptosis facilitator (Bim), Noxa and Puma. The results of this analysis are presented in Figure 5. We determined that the relative expression of Bik was significantly upregulated 2.3-fold in P10 S334ter-4 Rho retinas compared with P10 SD retinas (1.24±0.14 in SD vs. 2.73±0.3 in S334ter-4 Rho, *P*<0.001). On P10, we also observed an increase in the relative expression of the Bim protein (1.7-fold; 0.83±0.05 in SD vs. 1.40±0.21 in transgenic retina, *P*<0.05). At the next two time points, Bim expression was upregulated 1.34-fold compared with controls (0.69±0.15 in SD vs 0.51±0.10 in S334ter-4 Rho, *P*>0.05) on P12 and 1.8-fold compared with controls on P15 (0.86±0.18 in SD vs. 1.54±0.11 in S334ter-4 Rho, *P*<0.01). The Noxa gene expression increased significantly in P12 and P15 S334ter-4 Rho retinas and was 2.8-fold and greater than 2-fold higher compared to that in age-matched SD retinas, respectively (0.82±0.12 in SD vs. 2.34±0.36 in S334ter-4 Rho at P12, *P*<0.0001 and 0.88±0.24 in SD vs. 1.87±0.19 in S334ter-4 Rho at P15, *P*<0.01). On P15, the relative expression of Bid and Puma in S334ter-4 Rho retinas were increased 2.4- and 2.3-fold, respectively, compared to that in SD retinas (0.68±0.11 in SD vs. 1.62±0.23 in S334ter-4 Rho, *P*<0.01 for Bid and 1.00±0.05 in SD vs. 2.30±0.5 in S334ter-4 Rho, *P*<0.001 for Puma).

The apoptotic protease activating factor 1 (APAF1) also induces apoptosis. Therefore, we analyzed Apaf1 gene expression during the progression of ADRP and determined that its expression was upregulated 1.9-fold in P12 transgenic retinas (0.62±0.01 in SD vs. 1.17±0.10 in S334ter-4 Rho, *P*<0.01) (Figure 5). This upregulation leads to the activation of caspase-dependent apoptosis, which was confirmed by the analysis of caspase-3 and -7 expression levels. The caspase-3 and -7 proteins are executioner caspases. We determined that the expression of caspase-3 was significantly upregulated 1.9-, 2.17- and 1.8-fold in P12, P15 and P18 S334ter-4 Rho retinas, respectively (0.7±0.1 in SD vs. 1.33±0.2 in S334ter-4 Rho, *P*<0.01, 0.69±0.21 in SD vs. 1.5±0.15 in S334ter-4 Rho, *P*<0.01 and 0.90±0.10 in SD vs. 1.63±0.22 in S334ter-4 Rho, *P*<0.05, respectively). Caspase-7 gene expression was upregulated 1.73-fold and almost 2-fold on P10 and P15, respectively (0.82±0.04 in SD vs. 1.42±0.15 in S334ter-4 Rho, *P*<0.05 and 0.99±0.07 in SD and 1.92±0.43, *P*<0.01 in transgenic rats, respectively).

### Mitogen-activated protein kinases 1 (Erk2) and 8 (Jnk) are involved in retinal degeneration in S334ter-4 Rho rats

**Figure 6:**
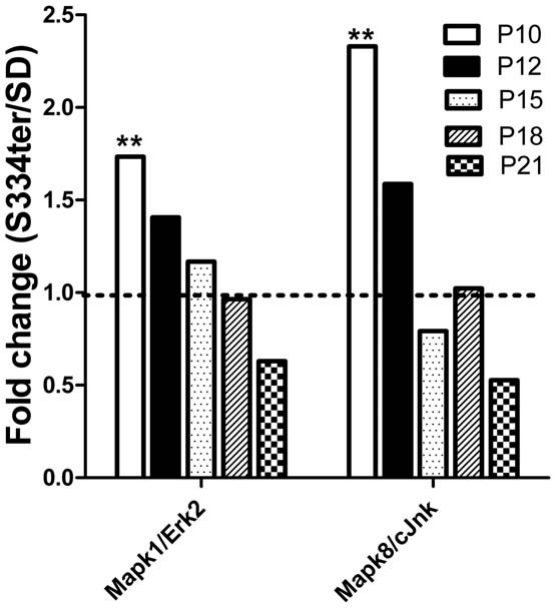
Relative expression of the MAPK1 (Erk2) and MAPK8 (JNK) genes in S334ter-4 Rho retinas. Relative gene expression in S334ter-4 Rho retina was measured on P10, P12, P15, P18 and P21 and a fold change was expressed as a ratio of S334ter-4Rho relative expression to SD relative expression. On P10, the Mapk3 (Erk1) and Mapk14 (p38) gene expression was upregulated 1.7- and 2.3-fold in S334ter-4 Rho retinas, respectively (*P*<0.01 for both). Starting on P12, their expression decreased with time.

A number of mitogen-activated kinases, such as Mapk8 (c-Jun N-terminal kinase 1) and Mapk14 (p38), play an important role in the ER stress response. Therefore, we included these and other members of the MAPK gene family, such as the extracellular signal-regulated kinases Mapk1 (Erk2) and Mapk3 (Erk1), in our study. RNA analysis of P10–P21 transgenic retinas demonstrated no significant difference in the relative expression of the Mapk3 and Mapk14 genes compared to control. In contrast, the expression of Mapk1 and Mapk8 was elevated by 1.7- and 2.3-fold, respectively, in the transgenic retinas (0.88±0.06 in SD vs. 1.5±0.20 in S334ter-4 Rho and 0.68±0.07 in SD vs. 1.60±0.3 in S334ter-4 Rho, respectively; *P*<0.01 in each case) on P10 and their expression subsequently decreased over time (Figure 6).

### pTen/Akt1 signaling is involved in retinal degeneration in S334ter-4 Rho rats

**Figure 7:**
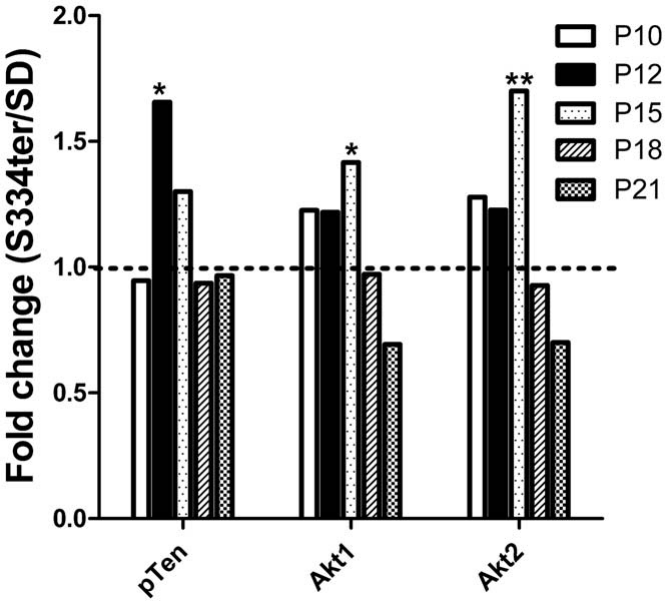
Relative expression of pTten, Akt1 and Akt2 genes in S334ter-4 Rho retinas. Relative gene expression in S334ter-4 Rho retina was measured on P10, P12, P15, P18 and P21 and a fold change was expressed as a ratio of S334ter-4Rho relative expression to SD relative expression. An approximate 1.65-fold increase in the relative expression of the pTen gene was observed (0.80±0.13 vs. 1.5±0.2, *P*<0.05) in P12 transgenic retinas. Subsequently, the expression of this gene decreased to WT levels. In contrast, the expression of the Akt1 and 2 genes increased after P12 and reached a peak on P15. The Akt1 gene expression was 0.84±0.15 in SD retinas vs. 1.40±0.13 in S334ter-4 Rho retinas, *P*<0.05. The Akt2 gene expression was 1.03±0.17 in SD vs. 1.70±0.15 in P15 S334ter-4 Rho, *P*<0.01.

A recent study by Hu and colleagues demonstrated a critical role for the endogenous Akt and MEK1/ERK pathways in counteracting ER stress and proposed that the endogenous Akt/IAPs and MEK/ERKs control cell survival by resisting ER stress-induced cell death signaling [24]. Figure 7 shows the progressive changes in Akt1/2 gene expression in S334ter-4 Rho rats during retinal degeneration. On P12, we observed the elevation of the tumor-suppressor gene pTen that catalyzes the dephosphorylation of the Akt gene leading to the inhibition of the Akt signaling pathway. An approximately 1.65-fold increase in the relative expression of the Pten gene (0.80±0.13 in SD vs.1.52±0.2 in S334ter-4 Rho, *P*<0.05) was observed. Subsequently, pTen expression decreased to the level of the WT samples. In contrast, the expression of the Akt1/2 genes increased after P12 and reached a peak on P15 in S334ter-4 Rho rats. The Akt1 gene expression increased by 1.65-fold (0.84±0.15 in SD vs. 1.39±0.13 in S334ter-4 Rho rats, *P*<0.05) and the Akt2 gene expression increased 1.7-fold (1.032438±0.17 in SD vs. 1.71±0.15 in S334ter-4 Rho, *P*<0.01).

### ER-mitochondrial cross-talk in S334ter-4 Rho retina detected by the elevated calpain activity and the cytosolic release of apoptotic inducing factor 1

The physical and functional interactions between the ER and the mitochondria occur throughout their networks. The molecular foundations of this cross-talk are diverse, and Ca^2+^ is an important signal that these organelles use to communicate. A recent study by Bravo et al. [25] demonstrated that during the adaptive phase of ER stress mitochondrial events are already underway before the appearance of cell death. The ER-induced Ca^2+^ release may facilitate the permeabilization of the mitochondrial membrane through the activation of the mitochondrial permeability transition (MPT) pore. In addition, Ca^2+^ release from the ER activates Ca^2+^-sensitive cytosolic enzymes, which may control the distribution and activity of Bcl-2 proteins and calpains or modulate the expression of apoptosis regulatory proteins.

**Figure 8:**
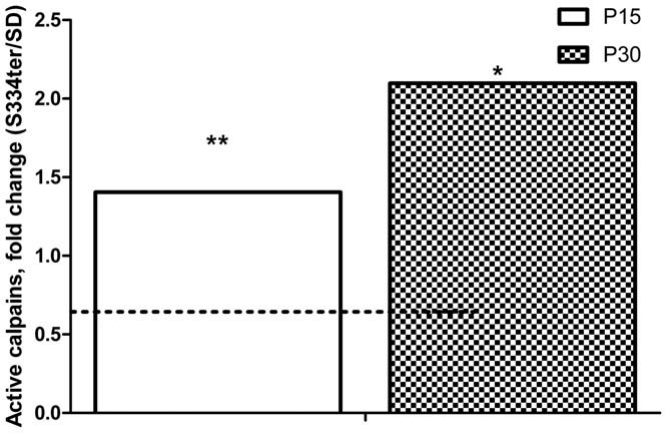
Activation of calpains in S334ter-4 Rho retinas. In P15 and P30 S334ter-4 Rho retinas, we observed an activation of calpains in the cytosol measured by the fluorescence intensity emitted from the calpain substrate Ac-LLY AFC. On P15 and P30, there was a 1.4- and a greater than 2-fold increase in activated calpains in S334ter-4 Rho retinas compared with SD retinas, respectively (2.24±0.09 in SD vs. 3.1±0.127 in S334ter-4 Rho, *P*<0.01 and 1.287±0.27 in SD vs. 2.7±0.04 in S334ter-4 Rho, *P*<0.05, respectively).

**Figure 9:**
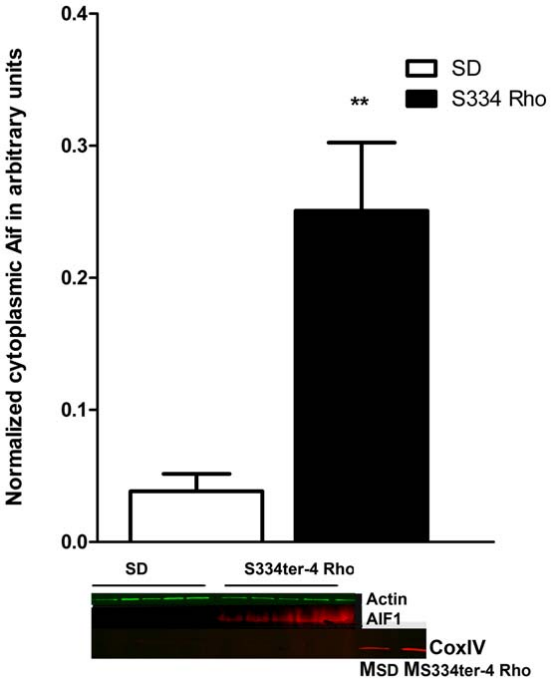
Release of AIF1 from S334ter-4 Rho mitochondria. Analysis of isolated cytosolic fractions of P15 S334ter-4 Rho retinas determined that the concentration of the cleaved form of Aif1 (48 kD) was 6.5-fold higher compared with the WT cytosolic samples (0.038±0.01 in SD vs. 0.25±0.05 in S334ter-4 Rho, *P* = 0.004). Images of western blot detecting the AIF1 and COXIV proteins are shown. Detection of COXIV protein was observed only in SD (Msd) and S334ter-4 (MS s334ter-4) retinal mitochondrial fractions.

Therefore, we investigated calpain activation in this study. Figure 8 demonstrates that compared with SD there is a 1.4-fold and a greater than 2-fold increase in activated calpains on P15 and P30 in S334ter-4 Rho retinas (2.24±0.09 in SD vs. 3.1±0.127 in S334ter-4 Rho, *P*<0.01 and 1.287±0.27 in SD vs. 2.7±0.04 in S334ter-4 Rho, *P<*0.05, respectively). To further understand if the activation of calpains triggers the MPT pore in S334ter-4 Rho retinas resulting in mitochondria-associated apoptosis, in P15 retinas we analyzed the mitochondrial release of the Apoptotic Inducing Factor 1 (AIF1) (Figure 9) and cytochrome *C*, which form an apoptosome with caspase-9 and apoptotic peptidase activating factor 1 (Apaf1) to activate caspase-induced apoptosis. In S334ter-4 Rho retina, we observed a 3-fold increase in the cytosolic AIF1 compared to SD. However, in P15 S334ter-4 Rho retina we did not detect any difference in the levels of cytochrome *C* compared to SD. In contrast, the relative expression of Apaf1 was upregulated (Figure 5).

Activation of calpains provokes cleavage of caspase-12. Therefore, we analyzed caspase-12 activity in transgenic retina and found that the level of cleaved caspase-12 was elevated in S334ter-4 Rho rats on P15 compared to control over 2-fold and was 0.05±0.007 in SD vs. 0.12±0.02 in S334ter-4 Rho, *P* = 0.014 on P15. However, further analysis of S334ter-4 Rho retina by western blot and caspase-12 fluorometric activity assay showed that the activity of ER-associated caspase-12 was declined in a time-dependant manner (data not shown). Comparison of the level of active protein kinase C (PKC), that has been earlier reported to inhibit the caspase-12 activity [26], did not reveal a difference between groups.

Knowing that calpains trigger the cleavage of AIF1 in the mitochondria resulting in its translocation to the cytoplasm, we analyzed the cleaved AIF1 protein (figure 9). There was a 6.5-fold increase in the levels of cleaved Aif1 in the cytoplasmic fraction of S334ter-4 Rho compared to SD photoreceptors on P15 (0.038±0.01 in SD vs. 0.25±0.05 in S334ter-4 Rho, *P* = 0.004).

### Time-dependent decline of Crx and Nrl transcription factors in the S334ter-4 Rhophotoreceptor

**Figure 10:**
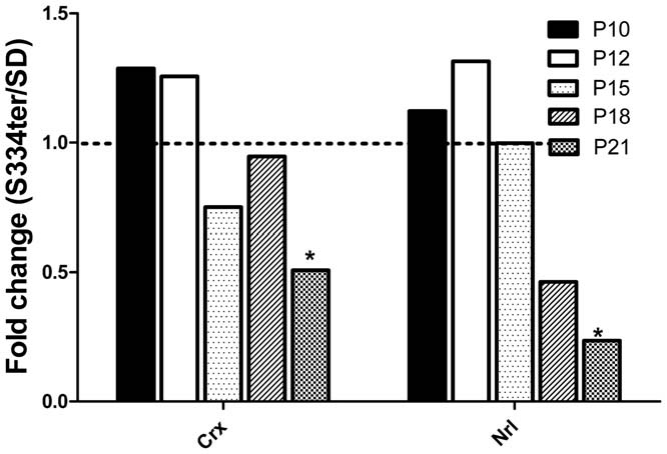
Relative expression of the Crx and Nrl transcription factors in S334ter-4 Rho retinas. Relative gene expression in S334ter-4 Rho retina was measured on P10, P12, P15, P18 and P21 and a fold change was expressed as a ratio of S334ter-4Rho relative expression to SD relative expression. The qRT-PCR analysis of S334ter-4 Rho retinas revealed that the expression of the Nrl and Crx transcription factors decreased in a time-dependent manner in P10-P21 retinas. A significant reduction in the expression of both transcription factors was observed in P21 retinas, the Crx gene expression was reduced 55% (*P*<0.05), and the NRL gene expression was downregulated 23% (*P*<0.05).

To estimate the massive loss of photoreceptor cells in S334ter-4 Rho retinas, we analyzed the relative expression of the NRL and CNX photoreceptor-specific transcription factors and determined that their expression patterns were altered during the progression of ADRP (Figure 10). The expression of the CRX gene was slightly higher in P10 and P12 S334ter-4 Rho retinas compared with SD retinas (0.95±0.04 in SD vs. 1.2±0.17 in S334ter-4 RHO, *P*>0.05 and 0.58±0.1 in SD vs. 0.70±0.12in S334ter-4 Rho, respectively). The expression of Crx decreased with time and was significantly lower in S334ter-4 Rho P21 retinas compared with SD retinas (1.10±0.11 in SD vs. 0.55±0.02 in S334ter-4 Rho, *P*<0.05). The levels of Nrl gene expression did not differ from WT levels during P10-P12. However, by P21, Nrl expression decreased dramatically in the S334ter-4 Rho retinas (1.00±0.21 in SD vs. 0.23±0.02 in S334ter-4 Rho, *P*<0.05).

## Discussion

Although retinal degeneration has many etiologies, they all lead eventually to photoreceptor cell death and blindness [27]. A number of investigators have dedicated their research to the elucidation of the mechanism of retinal degeneration in ADRP, and some of these studies have focused on ADRP progression in S334ter-4 Rho rats. For example, a recent publication by Kaur et al. highlighted the contribution of mitochondria-associated apoptosis [10] in which cytochrome *C* release plays an important role, and the contribution of non-apoptotic cell death, which involves calpain and PARP activation, to photoreceptor cell death and the degeneration of S334ter-4 Rho retinas. Despite the progression in our understanding of the cellular mechanisms leading to the decline of S334ter-4 Rho photoreceptors, the essential link that connects ER stress, UPR, and calpain activation with mitochondria-induced apoptosis has not been elucidated. In this study, we present a detailed investigation of the mechanisms of ER stress that lead to the activation of the UPR and apoptosis in retinas expressing mis-localized S334ter rhodopsin. We examine the status of the ER stress response and its correlation with pro-apoptotic gene expression over time (P10, P12, P15 and P21) and dissect the cellular mechanism of retinal degeneration during the development of a normal retina. Although gene expression during the maturation of WT retina is altered [28], [29], in this study, we focus on the relationship between gene expression and the progression of ADRP in transgenic rats expressing truncated S334ter rhodopsin.

The UPR is a conserved, adaptive cellular program that is activated in response to the accumulation of misfolded proteins in the ER [30]. Homeostasis in the ER may be compromised by a variety of stimuli including disturbances in redox regulation, calcium regulation, glucose deprivation, and viral infection. Oxidative protein folding depends upon the maintenance of adequate oxidizing conditions within the ER lumen and is achieved with the help of ER oxydoreductase (ERO1). ERO1 and PDI form the main pathway for protein disulfide bond formation in the eukaryotic ER. Therefore, the modulation of ERO1 activity is a component of a homeostatic feedback system in the ER that allows the cell to rapidly adjust to fluctuations in the ER redox environment and to maintain conditions that are conducive to oxidative protein folding [31]. We analyzed the expression of the Ero1 gene during the development and progression of S334ter-4 Rho photoreceptors on P10, P12, P15 and P21 and determined that there is a deficiency in Ero1 gene expression on P10, which is followed by a rapid over-expression of the gene on P12 (nearly a 2-fold increase) in transgenic retinas (Figure 2). After P15, Ero1 expression decreased dramatically, which reflects the demand for enzymatic protein folding in the cell and the adjustment of the ER to the redox potential. For example, during hypoxia, transcription of the Ero1 gene is dramatically induced through the activation of Hif1α [32], [33], suggesting that hypoxia negatively regulates the activity of many enzymatic pathways including Ero1. Therefore, in our experiments, the modulation of Ero1 gene expression suggests that there is an alteration in physiological oxygen tension and this could be considered as a key adaptive response to a hypoxia-induced Hif-1-mediated pathway (see below).

Recently, hypoxia-induced HIFf1 elevation has been studied in detail. HIF1a is a pivotal regulator of the cells' adaptation to hypoxia and is induced by hypoxia, growth factors, and oncogenes [34]. In addition, the elevated expression of HIF1a under hypoxic conditions is accompanied by the activation of ER stress genes, such as eIF2a [35], and by the increased generation of reactive oxygen species (ROS) that provide a redox signal for the induction HIF1α [36]. A number of investigators have proposed that retinal hypoxic preconditioning, which leads to HIF1α induction, morphologically and functionally protects retinal cells against light-induced retinal degeneration [37], [38]. Others have identified HIF1α as a protein that may be required (directly or indirectly) for the normal development of the retinal vasculature [39], suggesting that hypoxia is a part of normal retinal development. In addition, oxidative stress has been identified as an important contributor to retinal degeneration in a number of studies [40]. Thus, with respect to hypoxia, it is a logical assumption that Hif1α expression levels are also modulated in transgenic rats during retinal development. Therefore, it is not surprising that the level of Hif1a expression in S334ter-4 Rho retinas correlates with Ero1 transcription, increasing 1.5- and 2-fold on P10 and P12, respectively. The expression of Hif1α has not been examined previously in S334ter-4 Rho retinas. In this study, we demonstrated that hypoxic conditions leading to oxygen deprivation persisted, at least temporarily, in the developing S334ter-4 Rho retinas. The mechanism by which the Hif1α mRNA is induced in ADRP photoreceptors appears to be adaptive and is linked to mechanisms that maintain a homeostasis in the photoreceptor cells. In favor of this hypothesis, a rapid decline in Hif1α gene expression was observed in P15 S334ter-4 Rho retinas. Additional evidence supporting this hypothesis regarding the hypoxic status of transgenic retina comes from the analysis of the over-expression of the Nf-kB gene. Compared with Hif1α expression, Nf-kB over-expression was delayed until P15. The delay in the onset of Nf-kB over-expression may be associated at least in part with the modulation of Sod1 gene expression, which was over-expressed on P10. Recently, a direct link between the alternating expression patterns of both genes has been proposed [41].

SOD1 is a soluble protein that acts as a scavenger of superoxide converting it to molecular oxygen and hydrogen peroxide, and the SOD1 gene is considered the first line of defense against oxidative stress. The elevated expression of SOD1 has been associated with a number of neurodegenerative disorders, such as SALS and Alzheimer disease, suggesting that SOD1 upregulation is a pathological phenomenon [42]. Therefore, changes in Sod1 mRNA levels would indirectly reflect the increased accumulation of superoxide radicals in S334ter-4 Rho retinas. Although the Sod1 gene was over-expressed in P10 retinas, a dramatic drop in the expression levels of this gene was subsequently observed, which may be provoked by the induction of Nf-kB expression. The transient over-expression of Sod1 in S334ter-4 Rho retinas may be a result of the activation of the hydrogen peroxide-responsive element within the Sod1 promoter by H_2_O_2_ [42], [43], which has been shown to play a protective role in oxygen-deprived dopaminergic neurons in the rat substantia nigra [44]. Alternatively, an adaptive mechanism in S334ter-4 Rho photoreceptors that manages the stress by over-expressing a powerful antioxidant enzyme may be involved. As discussed above, the data indicate that the imbalance in ER homeostasis in S334ter-4 Rho retinas is created by hypoxic preconditions that lead to the induction of Ero1, Hif1α and Nf-kb gene expression in P10–P12. Therefore, we next investigated if the modulation of these genes provokes the activation of the UPR in S334ter-4 Rho retinas.

We analyzed the expression profiles of the following proteins: the ER resident chaperone proteins, such as calnexin (Cnx), Hsp40/Dnajc10, and Grp78/Bip; activating transcription factors Atf4, Atf6, and Xbp1; Gadd153/Chop, eIF2α (eukaryotic translation initiation factor 2α) and ERAD (ER-associated degradation) genes, such as Edem1, Edem2, Derl1, Derl2, and Hrd1 (Synovalin). The data suggest that during the development and progression of the ADRP retina, the expression of the majority of these ER stress gene is modulated. For example, the expression of Cnx was dramatically increased 1.5-fold and greater than 2-fold in P10 and P12 transgenic retinas, which paralleled the expression pattern of the Ero1 gene. The Cnx gene is an important component of the ER, where it is involved in the maintenance of ER protein homeostasis and participates in the folding and assembly of nascent glycoproteins and aids their transport out of the ER quality control system. Therefore, a high expression of the Cnx gene reflects a high demand for protein folding in the ER. One of these ER proteins may be an aberrant rhodopsin, which is rescued by the over-expression of the Cnx chaperone protein [45].

Further analysis of the ER chaperones Hsp40/Dnajc10, and Bip demonstrated that there is increase in the levels of Hsp40. Recently, a model of the Hsp40-mediated ERAD pathway has been proposed [46]. According to this model, the Hsp40 protein accelerates the ERAD pathway by reducing the number of incorrect disulfide bonds in misfolded glycoproteins that are recognized by EDEM1. ERAD substrates that are released from CNX are recruited by EDEM1 to the C-terminal cluster of HSP40. Therefore, an increase in Cnx suggests that there is a correlation between these synergistically working genes, which is confirmed by our experiments (Figure 2). Similar to the expression patterns of the Cnx gene, Hsp40 gene expression was also elevated 1.8- and 2-fold in P12 and P15 retinas, respectively (Figure 2), whereas the expression of Edem1 as Edem2 in the transgenic retinas (data not shown) was not significantly different from the SD retinas. This result suggests that in S334ter-4 Rho retinas, there is a high demand for the chaperoning assistance of Hsp40.

Another component of the ERAD pathway is the BIP (GRP78) protein. BiP binds to the J domain of Hsp40 in an ATP-dependent manner and transfers ERAD-targeted substrates to the retrotranslocation channel upon ATP hydrolysis [46]. In addition to participation in the ERAD system, BiP is an up-stream marker in the ER stress pathway and is the first line of defense in a compromised ER. This protein activates three independent UPR pathways, PERK, ATF6 and IRE1. RNA analysis of the S334ter-4 Rho and SD retina samples demonstrated that the increase in Bip expression correlated with the expression of Cnx and Hsp40/Dnajc10, reaching a peak on P15 with a 2-fold increase in the S334ter-4 Rho retinas. Western blot analysis was used to confirm the increased production of the BiP protein providing proof of the elevation of the BiP protein in S334ter-4 Rho rats.

The PERK pathway is activated in P12 transgenic retinas, which was evident from the upregulation of the eIf2a and Atf4 genes and elevation of peIF2α. In P15 retinas western blot analysis demonstrated the increased production of the peIf2α protein providing proof of activation of the PERK signaling pathways in S334ter-4 Rho rats. In addition, expression of the ATF6 (the ATF6 pathway) gene was increased steadily up to P15, after which they decreased to levels that were observed in the controls. Therefore, it is not surprising that the level of full-length pAtf6 (90 kD) and its cleaved form (pAtf6-50) in P15 S334ter-4 Rho retina were significantly increased. The level of Xbp1 (the IRE1 pathway) was also steadily increased up to P15. However, later in P21 retina expression of the Xbp1 gene was significantly reduced in transgenic retinas that could be a result of a decreased catalase expression, enhanced ROS generation, and the loss of mitochondrial MPTP after H_2_O_2_ exposure in S334ter-4 Rho photoreceptors [47]. In P15 S334ter-4 Rho retina, we observed increase in a spliced and unspliced forms of Xbp1 protein suggesting that the IRE pathways is activated. Despite the general decline in the Xbp1 gene expression in P21, the splicing of the Xbp1 mRNA was persistent in S334ter-4 Rho retina (figure 3 B).

Signaling through the PERK, ATF6 and IRE1 genes triggers pro-apoptotic stimuli during prolonged ER stress. However, these genes do not directly cause cell death, but they initiate the activation of downstream molecules, such as CHOP or JNK, which further push the cell down the path towards death. CHOP, a downstream marker in the UPR, is a pro-apoptotic protein that regulates the activity of genes including Bcl2, GADD34, ERO1 and TRB3 [48]. In our experiments, we demonstrated that Chop mRNA was elevated during ER stress in P12 S334ter-4 Rho retinas. The increase in Chop expression suggested that the adaptive phase of the UPR in the transgenic retinas initiated apoptosis, causing the S334ter-4 Rho photoreceptors to self-destruct. The over-expression of the CHOP protein was confirmed by western blot analysis suggesting that the CHOP protein is overproduced at transcriptional and translational levels. Therefore, in summary, we propose that all three UPR pathways are activated in S334ter-4 Rho retinas.

In general, the CHOP protein is post-translationally controlled by p38 MAPK (14). Although in our study, a significant difference in p38 expression was not observed, another MAPK 8 (JNK) was dramatically upregulated 1.6-fold in P10 transgenic retinas. This JNK protein is a stress-activated protein kinase that regulates apoptosis through the induction and/or post-translational modification of BH3-only proteins and plays a central role in setting the apoptotic cascade in motion. Evidently, in S334ter-4 Rho photoreceptors, the upregulation of the JNK gene is associated with the recruitment of c-JNK via the IRE1 pathway through TRAF2-c-JNK-ASK1. In support of this hypothesis, we observed the activation of the Ire1 pathways in P12 S334ter-4 Rho retinas. The expression of Xbp1 in P12 transgenic retinas was higher when compared with the control suggesting that the Xbp1 transcriptional factor is required by the elevated production of JNK. Another rationale for the increase in JNK expression is associated with the activation of Bim, Bak and Bax proteins [48].

Regarding the pro-apoptotic Bax/Bak BH3-only proteins, it is important to note that their relative expression did not change significantly in the transgenic retinas compared with the control retinas. This observation implies that the post-translational phosphorylation of BAX/BAK proteins is primarily a result of the increase in Jnk expression. In addition, in the developing WT retina, apoptosis appears to initiate the downregulation of Bax and Bak, which are key initiators of the caspase-dependent pathway [49]. The BH3-only BID protein participates in an extrinsic apoptosis that may occur in cone photoreceptor cells [50]. Because this BID protein is considered a component of caspase-8-induced apoptosis, the increase in its expression during P10-P15 may be associated with the elevated gene expression and activation of JNK that eventually cleaves BID into a novel form called tBID. This observation suggests that beginning on P10, JNK may be involved in TNF-mediated caspase-8 activation resulting in the activation of the BID protein followed by mitochondrial-associated apoptosis [51]. However, further investigation is required to confirm this hypothesis.

In general, we determined that other members of the BH3-only family of proteins are involved in retinal degeneration in S334ter-4 Rho rats. Thus, Bik (Bcl2-interacting killer) protein, which is a novel death-inducing protein, is over-expressed significantly in P10 retinas. The higher demand for the Bik protein in transgenic retinas may correlate with the changes in Bcl-xl gene expression. Again, in our study, a modulation of Bcl-xl gene expression in transgenic retinas was not observed. An alternative explanation for the increase in production of the BIK protein is that the elevated expression of the p53 gene in S334ter-4 Rho retinas promotes Bik mRNA expression [52]. In support of this hypothesis, we observed an increase in relative expression of other p53-induced proteins, such as Noxa and Puma. In P12–P15 S334ter-4 Rho retinas, the levels of Puma and especially Noxa (3-fold increase) are dramatically increased. Following the binding to anti-apoptotic proteins and the activation of Bax/Bak, PUMA-induced apoptosis proceeds through a typical mitochondrial pathway [53]. Therefore, we assume that on P12, the over-expression of Puma associates with the mitochondria membrane permeabilization transition pore (MPTP), which eventually leads to the cleavage and release of the AIFf1 protein and to the activation of caspase (see below). In addition, the increase in Noxa expression correlates with the upregulation of the Hif1α gene, which controls the expression of Noxa, on P10 [54]. Both Bid and PUMA trigger the mitochondrial apoptotic pathway leading to cytochrome *C* and AIF1 release from the mitochondria as demonstrated in our study (Figure 9).

The expression of the BH3-only Bim protein was elevated from P10 to P15 in S334ter-4 Rho retinas. The BH3-only BIM protein is an important initiator and regulator of the intrinsic pathway because BIM interacts with anti-apoptotic Bcl-2 proteins and the multidomain pro-apoptotic effector proteins BAX and BAK [55]. Because Bcl-2 expression was not modified, the increase in Bim expression may be associated with the upregulation of the Jnk pathway or the downregulation of the pro-survival Erk2 pathway in transgenic retinas. Recently, links between Bim and cJnk and between Bim and Erk signaling have been established [55]. Therefore, a decline in the expression of pro-survival Erk2 in P10 to P21 could regulate the Bim gene expression in S334ter-4 Rho retinas. An additional study has revealed that the level of Bim mRNA is positively regulated by C/EBPa and CHOP following ER stress [56], and this finding is in agreement with our results demonstrating an increase in CHOP expression in S334ter-4 Rho retinas.

Proteasomal degradation and autophagy are the two main mechanisms that control protein clearance in the cell. Unlike proteasomal degradation, autophagy degrades soluble and aggregated proteins. The molecular mechanisms responsible for the regulation of autophagy have not been completely elucidated; however, a recent study has demonstrated that severe hypoxia may lead to ER stress and may induce ATF4-dependent autophagy through LC3 as a survival mechanism [57]. In a study by Wang et al., the over-expression of KDEL (ER resident) receptors also activated autophagy [58]. It is apparent that the upregulation of the UPR genes increases the expression of KDEL receptors on the ER and this could promote autophagy in S334ter-4 Rho photoreceptors. The expression of lysosomal-associated membrane protein 2 or Lamp2 was induced significantly on P10. Our results are in agreement with the study of hypoxia-induced Lamp2 activation [59] in which the authors have proposed that hypoxia induces a high turnover of autophagic generation and degradation in cells.

The activation of calpains in transgenic retinas has been demonstrated [10]. Kaur et al. have shown that in S334ter Rho line 3 (a more rapidly degenerating line), the activation of calpain 3, which was measured using an *in situ* enzymatic assay on unfixed cryosections reaches a peak on P12. In our study of the S334ter Rho line 4 (a slower degenerating line), we discovered that on P15 the activation of calpains (1 and 2) is already pronounced (2-fold increase) and progresses along with retinal degeneration until P30. Our finding correlates with the study by Kaur et al. proposing that the proteolytic activity of calpains persists at times when the nuclear DNA has already disintegrated [10]. In agreement with these data, we found that the caspase-12 protein was cleaved in P15 S334ter-4 Rho retina as a result of activated calpains. Later, however, its activity measured in P21 and P30 S334ter-4 retinas was diminished. Evidently, transient activation of caspase-12 in P15 retina is sufficient to trigger the ER stress-associated apoptosis to contribute to a self-destructive program in S334ter-4 Rho photoreceptors. In addition, it has been proposed that caspase-12 is not required for caspase-dependant ER stress-induced apoptosis [60].

Therefore, we proposed that active calpains, together with the BH3-only proteins, Noxa, Puma, Bik, and Bid, compromised the MPTP in S334ter-4 Rho retinas and control a mitochondria-induced apoptosis. In support of this hypothesis, we detected the translocation of cleaved AIF1 from the mitochondria to the cytosol in S334ter-4 Rho retinas on P15. This data suggests that the S334ter-4 Rho mitochondria experience MPTP events that provoke caspase-independent apoptosis. To our knowledge, this is the first demonstration of AIF1 release from S334ter-4 Rho mitochondria. Meanwhile, in contrast to the study by Kaur et al. [10], the activation of caspase-dependant apoptosis through cytochrome *C* release from the mitochondria was not detected in our experiments. We did not observe difference in cytochrome *C* release between SD and S334ter-4 Rho mitochondria. However, this discrepancy between our study and the study by Kaur et al. can be explained by differences in the experimental approaches. Kaur et al. performed the analysis using fixed cryostat retinal sections, whereas we analyzed protein cytoplasmic fractions in which we had confirmed the absence of mitochondrial protein contamination.

Although we did not observe the cytosolic release of cytochrome *C* from mitochondria, an increase in the Apaf1 gene expression was detected suggesting the caspase-dependent activation of apoptosis. It is possible that the induction of Apaf1 expression in S334ter-4 Rho retinas is related to the upregulation of the p53 gene that controls APAf1 [61], Bik, Noxa and Puma. Therefore, p53 gene expression and the translocation of p53 to the mitochondria during the progression of ADRP should be examined in S334ter-4 Rho retinas. Despite studies demonstrating that retinal degeneration in rd1 mice occurs independent of p53 [62], others have demonstrated that the p53 gene plays a role in the regulation of photoreceptor apoptosis in inherited retinal degeneration [63], [64].

The expression of photoreceptor-specific transcription factors Nrl and Crx declined steadily in S334ter-4 Rho retinas between P10 and P21 and was reduced significantly in P21 retinas. These results suggest that in addition to the progressive collapse of photoreceptors in S334ter-4 Rho retinas, the transcriptional inhibition of Nrl and Crx may also take place. For example, it has been proposed that the over-expression of leukemia inhibitory factor (LIF), which is highly induced in developing ADRP mice retinas that express a mutant rhodopsin protein [65], reduces Crx and Nrl-dependent transcription [66]. Another explanation of the transcriptional inhibition of the Nrl and Crx transcription factors is linked to the inhibition of histone deacetylases (HDAC) that are diminished during retinal degeneration [67] and affect the RNA levels of these genes [68]. Apparently, the level of Hdac expression is modified in S334ter-4 Rho retina. In support of this hypothesis we observed the elevation in Apaf1 gene expression (Figure 5) that has been proposed to depend on the Hdac gene expression [61]. The future study of HDAC expression would also shed light on the upregulation of the Apaf1 gene in S334ter-4 Rho photoreceptors.

Our results describe mechanisms by which ER stress may be involved in the retinal pathology of S334ter-4 Rho rats, and how ER stress may be connected to mitochondrial dysfunction (Fig.S1). During hypoxia, the ER homeostasis in S334ter-4 Rho photoreceptors is compromised, which causes the activation of the UPR. The persistence of the UPR in S334ter-4 Rho photoreceptors leads to the upregulation of caspase-12 and BH3-only pro-apoptotic proteins, that together with calpains, induce MTPT. Our study and several other studies, have demonstrated that ER stress- and mitochondria-induced apoptosis culminate in the activation of caspase-3 in S334ter-4 Rho retinas. We believe that the activation of both ER stress- and mitochondria-originated apoptotic signals occur at approximately the same time (P12–P15) during retinal development in S334ter-4 Rho rats. In favor of this hypothesis, the expression of pro-apoptotic Bcl2 genes was significantly elevated in P12. Future experiments have to be conducted to establish a direct link between activation of the UPR and MPTP in S334ter-4 Rho rats. We also demonstrate that the relative expression of the UPR, pro-apoptotic, and oxidative-related genes in S334ter-4 Rho retinas have a temporal progression between P10 and P18. It is apparent that once triggered, cell death is executed rapidly and even the temporal expression of some genes in the P10–P15 retinas leads to apoptotic cell death. It is important to emphasize that in addition to caspase-dependent apoptosis occurring in S334ter-4 Rho photoreceptors, a caspase-independent pathway is induced by the release of AIF1 from the mitochondria. A study by Hong et al. has demonstrated a direct link between the release of the AIF1 factor from the mitochondria and the over-activation of PARP-1 [69] suggesting that our observation of AIF1 release could be considered as additional proof of a caspase-independent pathway that occurs simultaneously in photoreceptor cells. However, additional studies are needed to determine if AIF1 release contributes to the proposed non-apoptotic cell death in S334ter Rho photoreceptors [10].

Our findings indicate a number of genes that are potential therapeutic targets for ADRP gene therapy in S334ter Rho photoreceptors. This list includes but is not limited to Bik, Bim, Noxa, Puma and Bid proteins, calpains and caspase-12 proteins. Clearly, further studies are required to shed more light on the mechanisms involved in the induction of apoptosis, such as knocking down the expression of these genes in S334ter Rho retinas.

## Materials and Methods

### Ethics statement

The animal protocol was carried out with approval from the Review Board for Animal Studies at the University of North Texas Health Science Center (Approval Number # 2009/10-46-AO5) and in accordance with the guidelines of the Association for Research in Vision and Ophthalmology Statement for the Use of Animals in Ophthalmic and Vision Research. All efforts were made to minimize the number and the suffering of the animals used.

### Animal model

Homozygous S334ter rhodopsin transgenic rats (line 4) were maintained in the UNTHSC housing facility and were bred with WT Sprague-Dawley (SD) rats to generate heterozygous S334ter-4 Rhodopsin rats. Therefore, the SD rats were used as WT controls in our experiments. The animals were sacrificed on P10, P12, P15, P18 and P21 for RNA and protein analyses. All rats were maintained in specific pathogen-free (SPF) conditions with a 12-hour light and 12-hour dark daily cycle.

### RNA preparation and Real-Time PCR Analysis

Retinas from SD and S334ter-4 rats at P10, P12, P15, P18 and P21 of development were isolated. Total RNA was isolated from the individual retinas from each strain using an RNeasy Mini kit (Qiagen, Valencia, CA) (P10, N = 4; P 12, N = 6; P15, N = 5, P18 N = 6; P21, N = 6). Using individual retinal extracts of SD and S334ter-4 Rho retinas and a high capacity cDNA Reverse transcription Kit (Applied Biosystems); two cDNAs were prepared from each RNA sample. Each cDNA (10 ng) was subjected to qRT-PCR using Applied Biosystems TaqMan assays on 96-well plates (validated for each selected gene) on a One Step Plus instrument (Applied Biosystems, Foster City, CA) to compare the number of cycles (Ct) needed to reach the midpoint of the linear phase. All observations were normalized to the GAPDH housekeeping gene. The replicated RQs (Relative Quantity) values for each biological sample was average. Biological samples from each strain were used for the qPCR data analysis.

A semi-quantitative RT-PCR analysis of spliced Xbp1 was performed as described [70]. Quantification of the spliced portion of the XbP1 cDNA was performed by obtaining ratio of spliced Xbp1 to normalized unspliced Xbp1.

### Retinal protein extract for Western blot analysis

Retinal protein extracts were obtained from dissected retinas by sonication in a buffer containing 25 mM sucrose, 100 mM Tris-HCl, pH = 7.8, and a mixture of protease inhibitors (PMSF, TLCK, aprotinin, leupeptin, and pepstatin). The total protein concentration in right and left retinas from individual rat pups was measured using a Biorad protein assay, and 40 µg of total protein was used to detect individual proteins. The detection of proteins was performed using an infrared secondary antibody and an Odyssey infrared imager (Li-Cor, Inc.). Antibodies that detect the stress-induced phosphorylated proteins pPERK, pEIF2α, were from Cell Signaling (1∶1000). Antibody detected pATF6 (full land cleaved form) was from Imgenex (1∶1000). Anti-Grp78 and anti-CHOP (1∶1,000) were from Santa-Cruz Biotechnology; the anti-AIf1 and anti-caspase-12 antibodies (1∶1,000) were from Abcam and cytochrome *C* was from Santa-Cruz. β-actin was used as an internal control and was detected by the application of anti-β-actin antibody (Sigma-Aldrich).

### Isolation of cytoplasm and mitochondria from S334ter-4 Rho retinas

The isolation of the cytosolic fraction from the individual retinas of five SD and five transgenic rats was performed using the Mitochondria Isolation Kit for Tissues (Thermo Scientific). The mitochondria were separated from the cytoplasm using the Dounce stroke method as recommended by the Termo Scientific manufacturer. The protein concentration of each fraction was determined using a Biorad protein assay. To confirm the absence of mitochondrial contamination in the cytoplasmic fractions, a western blot was probed with CoxIV antibody (Abcam).

### Calpain activity assay

The detection of calpain activity was performed using the Calpain Activity Assay kit from BioVision in accordance with the manufacturer's recommendations and compared the activation of calpains in S334ter-4 Rho and SD retinal tissues. The detection of the cleavage substrate Ac-LLY-AFC was performed in a fluorometer that was equipped with a 400-nm excitation filter and 505-emission filter.

### Statistical analysis

All data were evaluated and plotted using GraphPad Prism5 software. We analyzed the results using Student's *t*-test for unpaired samples or a two-way ANOVA analysis of variance. All data are represented as the mean ± SEM. The P values that indicated statistical significance in experiments are “*” (*P*<0.05), “**”(*P*<0.01), and “***” (*P*<0.001).

## Supporting Information

Figure S1 **The role of ER stress in retinal degeneration in S334ter-4 Rho rats.** The ER stress caused by mis-trafficking of truncated rhodopsin protein contributes to the retinal degeneration in S334ter-4 Rho rats by inducing hypoxic conditions and compromising the ER homeostasis resulting in the activation of the UPR. The UPR in ADRP retinas is associated with the increased expression of the UPR markers, such as the eiF2 and Atf4 genes (the PERK pathway), the Atf6 gene (the ATF6 pathway) and the Xbp1 gene (the IRE1 pathway) and with elevated expression of BH3-only proteins. The BH3-only proteins, together with activated calpain, directly or indirectly control the integrity of the mitochondria through the translocation of active BAX/BAK, which causes an imbalance leading to the release of AIF1 from the mitochondria. Therefore, during ADRP progression, the ER stress signal communicates with the mitochondria to initiate the collapse of the S334ter-4 Rho photoreceptors.
(TIF)
