# Supplementary figures and images for: ER Stress in Retinal Degeneration in S334ter Rho Rats
Source: PLoS One. 2012 Mar 14;7(3):e33266. doi: 10.1371/journal.pone.0033266 (PMC3303830; doi:10.1371/journal.pone.0033266)

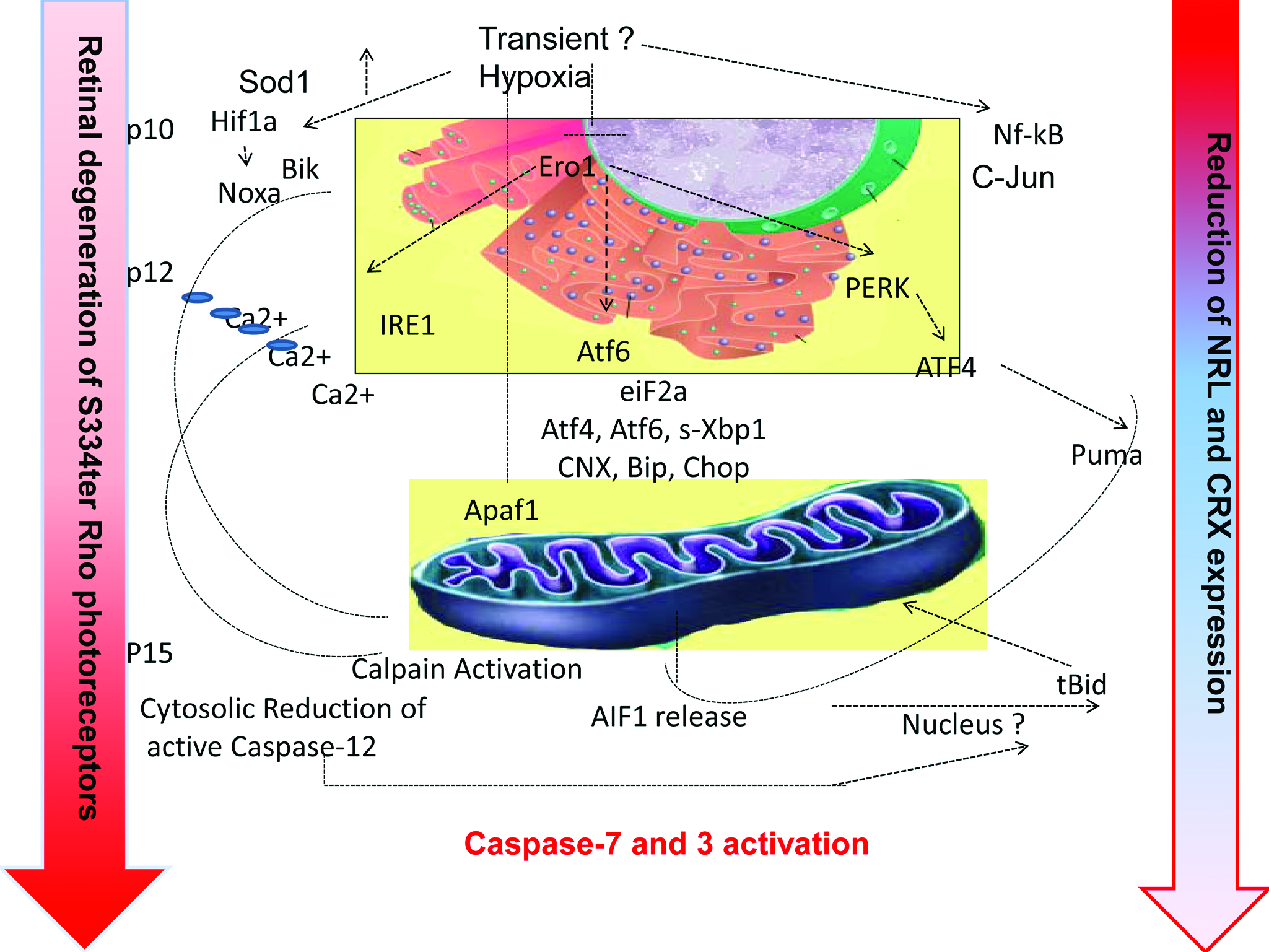

Supplement: Figure S1 — The role of ER stress in retinal degeneration in S334ter-4 Rho rats. The ER stress caused by mis-trafficking of truncated rhodopsin protein contributes to the retinal degeneration in S334ter-4 Rho rats by inducing hypoxic conditions and compromising the ER homeostasis resulting in the activation of the UPR. The UPR in ADRP retinas is associated with the increased expression of the UPR markers, such as the eiF2 and Atf4 genes (the PERK pathway), the Atf6 gene (the ATF6 pathway) and the Xbp1 gene (the IRE1 pathway) and with elevated expression of BH3-only proteins. The BH3-only proteins, together with activated calpain, directly or indirectly control the integrity of the mitochondria through the translocation of active BAX/BAK, which causes an imbalance leading to the release of AIF1 from the mitochondria. Therefore, during ADRP progression, the ER stress signal communicates with the mitochondria to initiate the collapse of the S334ter-4 Rho photoreceptors. (TIF) [file pone.0033266.s001.tif]
